# Navigating new sexual partnerships in midlife: a socioecological perspective on factors shaping STI risk perceptions and practices

**DOI:** 10.1136/sextrans-2019-054205

**Published:** 2020-02-10

**Authors:** Ruth Lewis, Kirstin R Mitchell, Catherine H Mercer, Jessica Datta, Kyle G Jones, Kaye Wellings

**Affiliations:** 1 MRC/CSO Social and Public Health Sciences Unit, University of Glasgow, Glasgow, UK; 2 Institute for Global Health, University College London, London, UK; 3 SEHR, London School of Hygiene & Tropical Medicine, London, UK

**Keywords:** sexual behaviour, qualitative research, prevention, social science

## Abstract

**Objective:**

Despite increases in STIs among those over 40, little is known about the social context of STI transmission among people experiencing relationship transition in midlife, and few sexual health promotion initiatives are targeted at this group. This study sought to identify factors shaping STI risk perceptions and practices among midlife individuals either contemplating or having sex with new partners following the end of a long-term relationship.

**Methods:**

Participants were purposively selected from respondents to Britain’s third National Survey of Sexual Attitudes and Lifestyles, using three eligibility criteria: aged 40–59, reported experience of the end of a marital or cohabiting relationship with an opposite-sex partner in the past 5 years, and willingness to participate in a qualitative interview. Qualitative data were generated via face-to-face interviews with 10 women and 9 men and analysed inductively using thematic analysis, with themes then organised using a socioecological framework.

**Results:**

Participants’ accounts of new sexual partnerships in midlife indicate that STI risk perceptions and practices are shaped by factors operating at multiple levels across the socioecological arena (individual, partnership, peers and communities, societal). Constraints on, and resources for, the navigation of sexual safety include self-perceived STI risk rooted in past rather than present circumstances; legacies of mistrust within former relationships; intersecting gender-age dynamics in negotiation of risk prevention strategies with new partners; peers and younger relatives’ influences on understandings of sexual risk and safety; postrelationship change in social networks that increase or mitigate vulnerability to sexual risk; age-related barriers to accessing condoms; and disconnection from safer sex messaging and services culturally coded as for the young.

**Conclusions:**

Improving sexual health among midlife adults requires age-sensitive interventions designed to address multilevel constraints, and harness positive influences, on the navigation of sexual safety at this stage of life.

**video abstract:** 

While younger people continue to account for the majority of STI diagnoses, increases in rates of STIs among people over 40 have been observed in high-income countries in recent years.^1–4^ In England, approximately 7% of new STI diagnoses in 2018 were among individuals aged 45–64 years old.^1^ Rising age at divorce^5^ and subsequent repartnering likely contributes to STI transmission during midlife among individuals not engaging in safer sex behaviours, such as condom use or STI testing. Identified barriers to STI risk prevention among midlife adults include low knowledge about STIs,^6^ prioritisation of intimacy above STI risks in new relationships,^7^ stigmatisation of STIs among older adults,^7^ reduced motivation to consider safer sex following removal of pregnancy risk due to menopause or permanent contraception,^7 8^ and barriers to sexual health discussions in general practice, including patient embarrassment to raise issues,^9^ general practitioners’ (GPs) fear of causing offence, and stereotyped assumptions that midlife and older people (especially women) are not sexually active.^10 11^

Despite prominent calls for greater attention to sexual health among those over 40,^12^ campaigns targeted at midlife and older adults remain rare in the UK^13^ and internationally,^14^ and there is a dearth of condom use^15^ or testing interventions aimed at these age groups. In order to inform and support these required initiatives, greater understanding is needed regarding the social context of STI risk and prevention among people either entering or contemplating new sexual partnerships at midlife. In particular, insights are required into how orientations to, and experiences of, risk prevention are shaped through interactions with sexual partners, peers, communities and broader society.

Drawing on qualitative data, this paper uses a socioecological framework to conceptualise the social context of STI risk perceptions and practices among people who have experienced the breakdown of a cohabiting/marital relationship with an opposite-sex partner in midlife (see figure 1). Modified socioecological models (MSEMs) have been used to depict multilevel and interacting factors influencing risk among populations vulnerable to STI and HIV, including men who have sex with men (MSM),^16 17^ women living in areas of socioeconomic deprivation^18^ and young people,^19^ but have not yet been used to characterise the social context of STI risk among midlife men and women experiencing transition from opposite-sex relationships. Enhanced understanding of these multilevel factors may help identify opportunities for targeted intervention.

**Figure 1:**
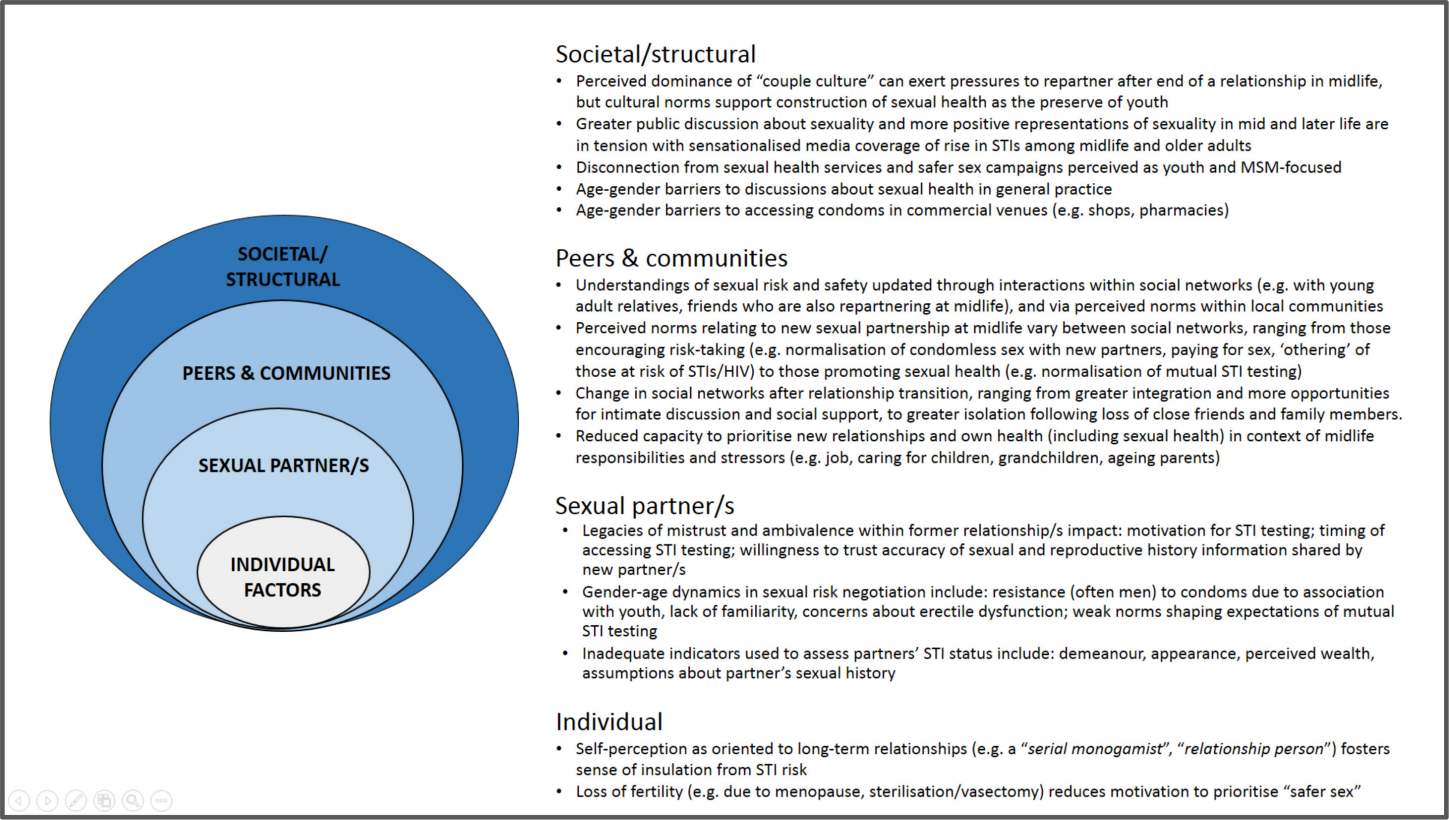
Modified socioecological model representing multilevel factors shaping STI risk perceptions and practices after the end of an opposite-sex relationship at midlife. MSM, men who have sex with men.

## Methods

Indepth interviews were conducted with a subsample of respondents to the third British National Survey of Sexual Attitudes and Lifestyles (Natsal-3). Full survey methods are described elsewhere.^20^

Participants eligible for this study were 323 women and 205 men aged 40–59 years (defined here as midlife) who reported having experienced the end of a cohabiting/marital opposite-sex relationship in the last 5 years. The social context of STI risk among individuals ending same-sex relationships was deemed to warrant a dedicated, culturally focused study. All Natsal-3 respondents were asked in the questionnaire to indicate willingness to participate in a follow-up interview (no topic defined); among those eligible for this study, 279 women and 176 men agreed. We drew a purposive sample guided by recency of participating in the Natsal-3 survey, roughly equal gender distribution and geographical spread across Britain.

Interviews were conducted with 19 individuals (10 women, 9 men) (see table 1). Our sample was socioeconomically and geographically diverse, spanning 14 counties in England and Scotland. Out of 19 participants, 14 reported a new sexual partner since their relationship breakdown; the other 5 all expressed some degree of openness to new sexual partnerships.

**Table 1:** Selected characteristics of participants (n=19)

	Women	Men
Age		
40–49	8	5
50–59	2	4
Legal relationship to ex-partner		
Divorced	8	6
Separated	1	1
Never married	1	2
New sexual partner/s since ex-partner		
Yes	6	8
No	4	1
Current relationship status (self-defined)		
In a long-term/committed relationship	4	6
Dating/seeing someone	1	1
Not in a relationship	5	2
Children		
Yes, living with participant most/all of the time	8	2
Yes, living with ex-partner most/all of the time	0	2
Yes, grown up and living independently	1	5
No children	1	0

Interviews were in participants’ homes, with written consent obtained from all participants. The topic guide explored perceived norms and experiences regarding new sexual relationships at midlife, conceptualisation and prioritisation of sexual health, safer sex strategies, and help-seeking for sexual health information and advice. Interviews were audio-recorded and professionally transcribed.

We conducted thematic analysis, guided by Braun and Clarke’s six-stage framework.^21^ We familiarised ourselves with data by rereading transcripts and writing case summaries. Two researchers independently generated open codes across a subsample of transcripts, with initial codes then reviewed, discussed and combined into potential themes. As we worked iteratively to define, name and check candidate themes, we recognised the value of using a socioecological framework to organise themes. We drew on MSEMs conceptualised at four levels,^22^ and separating sexual partners from other interpersonal relationships^19^ to group themes at four levels: individual, sexual partner, peers and communities, and societal/structural.

## Results

**Figure 2:**
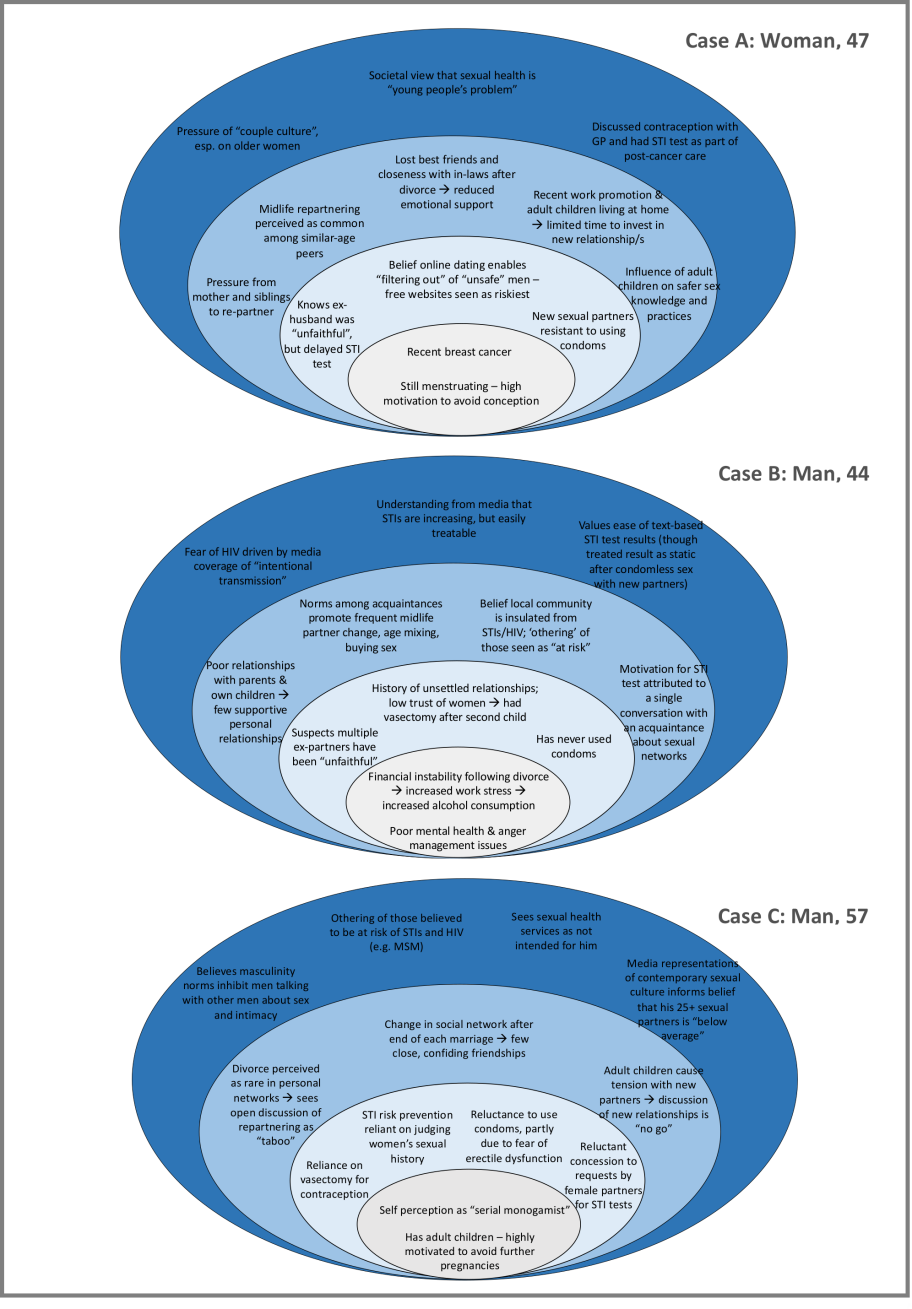
Examples of combinations of factors within three individuals’ accounts that contribute to perceptions and practices of STI risk and prevention after midlife relationship transition across multiple ecological levels (individual, sexual partner(s), peers and communities, structural/societal). For all participants, factors operated at each layer of social context, although individuals varied in the number of factors articulated at each level. For example, cases A and B articulated more factors (both constraints and resources) at the peers and communities level, in contrast to case C, where factors shaping risk prevention were mainly focused at the partnership level. Factors also interacted across levels to contribute to heightened vulnerability; for example, case A articulated inability to consistently negotiate condom use with new partners despite her desire to do so as being compounded by loss of social support after the end of her marriage, multilevel pressures to repartner and disconnection from sexual health services and campaigns perceived as youth-focused. GP, general practitioner.

Previously unpublished Natsal-3 data indicate 12.2% of all men and 8.9% of all women aged 40–59 reported at least one *new* opposite-sex partner in the past year. Among this subgroup, around half considered themselves to be not at all at risk of STIs or HIV, almost three-quarters reported condomless sex with new sexual partner(s) in the past year, while only around 1 in 20 reported having attended a sexual health clinic in the past year (see online supplementary appendix table 1). The current qualitative study builds from these quantitative findings to illuminate the multilayered social context of STI risk and prevention following midlife relationship transition, based on women’s and men’s accounts (figure 1 and table 2). Figure 2 illustrates how these levels of influence interrelate to produce a social ecology conducive to STI transmission, drawing on three case studies to demonstrate interplay between factors at different levels.

10.1136/sextrans-2019-054205.supp1 Supplementary data

**Table 2:** Extracts illustrating themes within participants’ accounts

Factors relating to/about individuals	**Self-identity regarding orientation to relationships.** “I’ve always been in long term relationships…it’s not been an issue, if you know what I mean? Like I’ve got mates that go out and sleep with everyone. Now, obviously they do use precautions, that lot, so they say. But it’s never been an issue. See, I’ve had the snip as well, so….” (Man, 45) ­ “When you sort of come out of your [marriage], your sort of sexual identity is from the 80s and you’ve been with one person for all that time you don’t think down that route at all […] you go through a sort of a 20 year period where AIDS and sexual health becomes something that people can talk about and you just think to yourself, oh my God, I’m glad I’m not in that category, y’know, I’m glad I know who I’m with and what’s he done and […] Y’know we’re both loyal to each other, we’ve been together for 20 years so if either of us had got AIDS we would have both had it by now, that sort of thing […] And you don’t worry about it because you don’t see yourself ever being with anybody else and then suddenly your marriage breaks up and you’re both with new people. But again, you’re not in relationships – or you don’t think you’re in relationships with people that have been around because it’s somebody else that’s been in a long-term marriage and you think ‘right, okay we’re sort of kindred spirits here, we’re in the same boat, there isn’t an issue.’” (Woman, 44)
Factors relating to/about sexual partners	**Legacy of mistrust and ambivalence within former long-term relationships.** Immediate testing in the context of suspected non-monogamy. “…as soon as I kind of finished with what’s-his-name I did actually go to the clinic, I had my smear test done and tested everything else [i.e. STIs], just wanted to make sure everything was clear because sometimes your mind can be thinking it overtime and thinking ‘oh he’s been sleeping with somebody else or whatever’ so you know, it is important to be careful.” (Woman, 43)“ ­ …when I split up with [ex-partner]– I think she’d slept with somebody else before we split up anyway – I went up to the hospital and had a [STI] test, like full MOT.” (Man, 44) ­ Delayed testing and ambivalence in the context of suspected non-monogamy.“ I did end up having tests and stuff but it wasn’t really because of that, I don’t think he [ex-husband] would be the type that would sleep around with lots of people, I didn’t think that because he’d done it with her [i.e. during their marriage] he must have slept with loads of different women because he wasn’t like that and I don’t think he intentionally went out to do it […] I didn’t think that I could catch something.” (Woman, 46) ­ **Gender-age dynamics in negotiation of safer sex with new partners.** Negotiating condom use. “[*describing recent experience using condoms for first time in twenty years*]…it was like being a kid again, yeah, because although you know what to do, that doesn’t help the situation, it is a passion killer and you fumble about. Don’t know, not a good experience, I don’t enjoy that.” (Man, 48) ­ “He [new sexual partner] didn’t worry about using a condom, y’know, he sort of come in with that mindset. It was just a case of ‘oh well I’ve had a vasectomy so you won’t have any children.’” (Woman, 44) ­ “Y’know, it is strange how you find how many men, especially my age group, don’t use condoms and you have to question them, y’know, are they going to use anything? You have to sort of initiate the conversation where it should be automatic, I think. But I got to the stage where I carried them because, yeah, well they didn’t have them and you don’t know, especially with online dating, how many of them do that […] I think it could be quite risky if I didn’t insist that we use condoms but I find that some men just don’t think they’re necessary […] They’d say, well you’re on the pill aren’t you? Well no, I’m not actually. *(Laughs)* […] And I don’t know if they seem to think getting a sexual disease is a young person’s problem. They seem to think because they sleep with older people a lot that it doesn’t matter and it does matter!” (Woman, 47) ­ Negotiating mutual STI testing. “My last girlfriend […] she was concerned about it [STIs), because after I’d broken up with my previous girlfriend, I had slept with a couple of women, so she actually wanted me to get a test and, in fact, I went and got a test. And I never said anything, but I was annoyed about it. She wanted me to do it because, perhaps she wanted to be sure, so I did it and showed her the clean bill of health, and off we went […] I think she could have taken my word for it, because I know the women I’d been with were very– not promiscuous at all, so I was kind of annoyed about it, really.” (Man, 57)
Factors relating to/about peers and communities	**Peer and community influences on understandings of sexual risk and safety.** Learning from similarly aged peers. “Well it’s interesting because the biggest rise in STDs is in the sort of 50, 45–50 plus age group, isn’t it? […].” *“So does that cross your mind about being a new risk group?”* “Yeah it does, it does. I mean, my friend and I, we’ve kind of had this conversation. We went on holiday and she did meet this guy, and we just got talking and she said she’d said to him ‘No condom, no sex, until you bring me that piece of paper telling me that everything’s alright’, and he did, he went and got the piece of paper for her, yeah […].” *“What did you think about that strategy?”* “Well it’s very sensible, very sensible […] you would be silly not to, wouldn’t you? It wouldn’t be very sensible not to, y’know, you just don’t know, you just don’t know.” (Woman, 58) ­ Learning from younger relatives. “I have conversations with my nephew…they all go to the GUM clinic regularly, and they go en masse…when I was first sort of yes I’m in another relationship and he was saying ‘now if you’re worried about getting condoms or anything or you want to go to the GUM clinic, come with me and I’ll get them for you’…I mean it’s nice to think that kids these days can be like that and it makes you sort of think oh it’s no biggie, y’know, you sort of get down with the kids, do what they do really, y’know?” (Woman, 43) ­ “I noticed a little sample bottle thing on the side and I said to him [son], ‘what’s that for?’ He goes, ‘oh I’m just going to be tested’ because he’d been seeing his girlfriend about six months and I go ‘why?’ and he goes, ‘well me and her have decided that as she’s on the pill that we won’t use condoms anymore so we’re just being tested first to make sure we’re both alright and then we’re not going to have condoms anymore’ and I just thought that was quite a responsible thing to do, of a boy of twenty he was at the time. So I suppose because he told me that I was sort of thinking well really, it’s a good way to think. It’s not just about getting someone pregnant, it’s about being safe.” (Woman, 47)
	**Change in networks of support and care after relationship transition.** Postrelationship changes in social network, greater social integration. “I’ve got a very good and strong network of friends and family around me […] four of my very close friends, we’re all in the same situation, we’re all single so a lot of my friends around me, one by one they’re all kind of separating […] and I don’t like it but unfortunately my friendship group and my ability to go out and enjoy myself has become more and more possible because more and more people are splitting up […].” (Woman, 48) ­ Reduced capacity to prioritise sexual health in the context of midlife stressors. “[*reflecting on sexual safety following discovery of ex-husband’s non-monogamy*]I didn’t think about it [own sexual safety] […] I was working and also having the kids and at first I didn’t think about it until the kids got a bit older and you have some more time for yourself and then you start thinking of things, yeah, then you start worrying.” *“What did you worry about?”* Getting something, ‘cos he [ex-husband] got crabs and then when I found out he was sleeping with men as well that was it…” (Woman, 46)
Factors relating to/about broader structures and society	Media landscape shapes understandings of dating, sex and sexual health at midlife. “I don’t want no sexual diseases or nothing like that.” *“And would you see that as a risk now for you?”* “Yeah, there’s a lot more of it about lately, y’know, or so you read in the papers and that. Everybody’s at it and a lot of sexual diseases about, so there’s more chance of getting one now than there was when me and my (ex)wife was younger.” (Man, 58) ­ “I’ve probably only slept with about 25 women in my life, which I think is actually quite low compared to young people, I’m guessing, what do I do know? But you think they’re at it all the time and with each other and everywhere, from what you read in the paper…” (Man, 57) ­ “…in your 40s you stereotype things on what you learn, and if you look at programmes that you’ve watched on telly in the 70s and the 80s, you take a lot of that on board and then you sort of, as I say, you think about the classified ads and the internet dating and you see all these things on the TV and as somebody older you think ‘oh no, I couldn’t possibly do that!’ But then loads of people do, so you think ‘oh fair play, if that’s the way it is.’” (Woman, 44) ­ Age-related barriers to accessing sexual health services. “I thought it [GUM clinic] was for dirty people, and when you went there you feel at ease actually ‘cos they don’t make you feel like you’re disgusting sort of thing, you know, the nurses and that, they don’t make you feel like you’re a tramp, I don’t know if that’s the right word but that’s how I, you do, you feel like it’s for young people who sleep around and stuff, but sometimes it’s really not, it could be just someone coming from one relationship to another and have caught something can’t it? And then I, they’re quite old they were, you know, well older than me some of them!” (Woman, 48) ­ Age-related barriers to accessing condoms in commercial venues. “You throw caution to the wind, y’know, I suppose with the no pregnancy risk. So yeah, you have to be mindful of that, so that means thinking about being prepared […] I mean you’ve got to be prepared, but I’m in my 50s, so what, I go into a shop and they’d be like ‘what are *you* doing buying condoms?’” (Woman, 58)

GUM, genitourinary medicine; MOT, Ministry of Transport.

### Individual

Although all participants described their own sexual risk as low, accounts of new sexual partnerships at midlife indicated a potential disconnect between actual and perceived risk, with many describing sexual encounters involving known risk factors (eg, condomless sex with new partner(s), lack of STI testing, lack of knowledge of partner’s STI status). Accounts of self-perceived low STI risk were strongly grounded in self-identity regarding one’s orientation to relationships (eg, assertions of being a ‘serial monogamist’ or ‘relationship person’), which appeared to foster a sense of perceived insulation from STI risk, especially when combined with a belief that the new partner(s) was similarly oriented. Loss of fertility (eg, due to menopause, sterilisation/vasectomy) appeared to also strongly affect motivation to use condoms, with several women and men expressing low prioritisation of ‘safer sex’ in the context of removed risk of pregnancy.

### Sexual partner(s)

#### Legacy of mistrust and ambivalence within former long-term relationships

Many participants described their willingness and ability to initially trust new sexual partner(s) as severely reduced due to the legacy of struggles and trauma in former relationships (eg, non-consensual non-monogamy, domestic violence, problematic alcohol use by partner). Scepticism about former partners’ monogamy was often cited as a motivation for a midlife sexual health check in theory, although not all who expressed doubt sought STI testing—a situation participants sometimes spontaneously accounted for by expressions of residual faith that their (now ex) partner would only have had sex with ‘low risk’ partners. Among those who had sought testing during midlife (five women, four men), timing between doubt of a partner’s monogamy and testing varied; while some reported seeking testing immediately on discovery (suspected or confirmed) of a partner’s non-monogamy, others described a lag between doubt and testing as they waited until later points, such as when the relationship had definitively ended, when planning to start dating or at the start of a new relationship.

#### Intersecting gender-age dynamics in negotiation of safer sex

Negotiation of sexual safety with new partners was clearly constrained by intersecting gender-age dynamics. Prior strategies for avoiding unwanted pregnancy were sometimes no longer relevant (eg, women’s reliance on a former partner’s vasectomy) and required renegotiation. Condoms were commonly described by women and men as embarrassing to discuss and use with new midlife partners for various reasons, including perceived association with youth, lack of familiarity after decades of condomless sexual activity and their perceived exacerbation of erectile dysfunction in new partnerships. Middle-aged men’s particular resistance to condom use was described by both men and women, presenting challenges for women’s insistence, and expressed resentment at having to assume sole responsibility for initiating their use. Accounts of STI testing in the early stages of a new sexual partnership were mostly attributed to requests (and sometimes insistence) by female partners, rather than at men’s own initiation. Where testing had occurred, results were sometimes characterised as static (eg, ‘having the all clear’), even after condomless sex with new partners in the interim. In the absence of normalised expectations of mutual STI testing, evaluation of the STI status of potential partners was often based on inadequate indicators, such as appearance, demeanour, perceived wealth, assumptions (rather than discussions) about their sexual history, duration between meeting and sexual activity, and among men, women’s willingness to engage in specific sexual practices (eg, anal intercourse).

### Peers and communities

#### Peer and community influences on understandings of sexual risk and safety

Peers and social networks were important informal systems shaping understandings of norms relating to sexual risk and safety at midlife, with participants commonly comparing their own experiences of dating, sex and risk negotiation with new partners with those of similarly aged, *known* others within their personal networks (eg, relatives, friends, colleagues), as well as perceived norms within wider local communities. Some participants described pressure from friends and family to repartner, while simultaneously being warned that sexual cultures had shifted since they were last ‘single’ and required new strategies to keep themselves ‘safe’. In some cases, friends and family were recruited to help assess the sexual risk associated with new (or potential) partners, although sometimes using unreliable indicators, such as the individual’s apparent interest and ease in integrating into new social networks. Exposure to younger relatives’ (eg, young adult children’s) own sexual health practices were an important context for updating knowledge and validating safer sex strategies, such as negotiating mutual STI testing in the early stages of a new partnership.

#### Change in networks of support and care after relationship transition

Change in social networks following relationship transition both increased and mitigated sexual risk among interviewees. For some, total loss of contact or growing emotional distance from formerly close friends and family members (eg, in-laws) fostered feelings of social isolation and reduced opportunities for social support regarding their personal lives. Some men described new patterns of socialising postdivorce/separation that increased vulnerability to sexual risk, including more time spent with casual acquaintances in social environments with community norms that encouraged high alcohol consumption, frequent partner change, age mixing, paying for sex, and ‘othering’ of those at risk of STIs and HIV (ie, believing themselves to be insulated from infection). By contrast, others (mostly women) described feeling more socially integrated after relationship transition, within both existing and newly forged social networks, and having more freedom to discuss intimate matters (including sexual relationships and health) with friends once they no longer felt bound by loyalties of discretion to former partners. Yet, in the context of competing midlife demands, several participants conveyed limited capacity to prioritise new personal and sexual relationships, or their own health (including sexual health); there was an evident tension between *needing* additional support at times of relationship transition and *being needed by others* (eg, children, ageing parents). Women especially described prioritising children’s emotional needs following their separation/divorce (including children’s resistance to parental repartnering) over their own.

### Structural/societal

Several features of the wider social and structural context appeared to contribute to understandings and experiences of navigating sexual safety at midlife. Greater public discussion about sex and sexual health information than in previous eras was broadly described as positive, as were increasing representations in mainstream and social media of sexuality among midlife and older adults, although not all portrayals were seen as helpful (eg, constructions of sexually active women as ‘cougars’). News stories were also credited with raising generalised awareness of rising rates of STIs among those over 40, yet some participants noted they did not relate to the extreme case examples commonly included in this coverage. In terms of healthcare, participants’ belief that ‘sexual health’ is coded as relating to young people extended to a perception of specialist sexual health services as youth-focused and therefore embarrassing to access as a midlife adult (although those who had generally reported positive experiences). Only one participant reported sexual health being discussed in general practice settings, although valued discussions about contraception and sexual function (eg, erectile dysfunction(ED), lubrication) had occurred during clinical encounters focused on treatment and management of other conditions (eg, cancer, diabetes). Barriers to accessing condoms in non-sexual health settings (eg, shops) included fear of ageist judgement, especially among women.

## Discussion

Despite awareness of rising prevalence of STIs among midlife adults, few participants in this study consistently used condoms with new partners, routinely sought STI testing or perceived themselves at risk of STI. A tendency to characterise one’s exposure to STI risk in relation to a self-identity grounded in former relationship status, rather than current circumstances, may shed some light on specific age-related factors contributing to the common underestimation of one’s STI risk.^23 24^ Yet barriers to STI prevention extended beyond individuals’ self-perceived risk, accumulating across layers of social interaction—with sexual partners, peers, communities and broader social structures—and coalescing to produce a social environment conducive to midlife STI transmission. At the sexual partner level, condomless sex was habitualised over decades of sexual activity with former long-term partner(s), strategies used to assess sexual safety of new partners were often inadequate (eg, assessments based on reputation), and conversational norms to enable risk negotiation were unresolved. A particular contribution of this study is elucidation of factors operating at the peers and communities level, where changing social environments following relationship transition could increase vulnerability by promoting sexual risk-taking, reducing opportunities for informal support about sexual relationships and health, and reducing capacity to prioritise one’s sexual health in the context of intensified caring responsibilities. At the structural level, barriers to STI prevention among midlife adults included perception that sexual health campaigns and services are not geared towards their age group—a sense of disconnection potentially exacerbated by the often-sensationalised tone of coverage about STI risk among midlife and older adults; in combination, these factors appear to conspire to construct a belief that sexual health services are necessary for ‘others’, but not oneself. Yet, alongside these multilevel constraints, resources for STI prevention also operated across multiple layers of social context, including increased social support at times of relationship transition, informal discussions about risk prevention with friends and family, and via exposure to sexual health-promoting behaviours among younger relatives, as also reported elsewhere.^6^

The strengths of this study include rich description and prioritisation of subjective meanings; use of a socioecological framework to highlight the multilevel social context shaping perceptions and practices; and a sample that included non-users of sexual health services. The limitations include potential social desirability bias within face-to-face interviews and exclusion of those acquiring new sexual partners concurrent with an ongoing marital/cohabiting relationship—a likely subgroup of midlife people experiencing new STI diagnoses. A parallel investigation with individuals who have ended a same-sex relationship in midlife would more fully expand understanding of factors shaping STI risk perceptions and practices among those over 40.

Given the compounding pressure on sexual health services in the UK, including increases in STIs and dramatic cuts to funding,^25^ careful thought must be given as to how to address unmet need for sexual health promotion among midlife adults with opposite-sex partners, while not jeopardising justifiable focus on groups at greatest risk of STI (eg, young people and MSM). These data suggest potential priorities and promising points of leverage. A possible unintended consequence of sexual health initiatives’ focus on younger people is perpetuation of the impression that these messages are irrelevant to older adults^26^—a suggestion our data appear to support. Age-specific campaigns may be needed to redress the disconnect to safer sex messaging that midlife adults contend with after the end of a long-term relationship. Over the past decade, a handful of UK-based national and local campaigns have aimed to raise awareness of STIs and encourage condom use and testing among midlife and older adults^27 28^; it is essential that promising efforts such as these are robustly evaluated with regard to their reach, acceptability and impact. Moreover, while campaigns encouraging condom use and testing among midlife and older adults are valuable, as with all age groups, knowledge alone is insufficient to ensure sexual safety. Interventions might also address age-specific constraints on risk prevention by equipping midlife adults with skills to negotiate condom use and mutual STI testing with new partners, including working to normalise conversations and address resistance in the context of age-related sexual dysfunction, absence of pregnancy risk, and often deep-seated legacies of mistrust and ambivalence within former relationships. Future interventions might seek to harness existing flows of sexual health information and positive influence within social relationships, such as midlife adults updating their understandings of sexual safety through discussion with friends and family, especially younger relatives.

In terms of services, participants’ accounts indicated virtual absence of GP-initiated discussion of sexual matters and a perception of specialist sexual health services as youth-focused. Previous research suggests preference for STI diagnosis and treatment in general practice among at-risk individuals not attending sexual health clinics.^29^ GP training may, therefore, be useful to challenge assumptions about the sex lives of midlife patients and equip them with confidence to raise discussions. At the same time, sexual health services may require support to redress their image as youth-focused and ensure they are meeting the needs of midlife patients. This might involve targeted efforts to increase availability and uptake of community-based condom distribution and testing.

In the context of an ageing population, with many people sexually active into later midlife, maintaining momentum for sexual health protection throughout the life course is crucial. Public Health England recently urged: ‘*No matter what age you are* […] if you have sex with a new or casual partner, make sure you use condoms and get regularly tested’.^30^ Meeting this target among midlife adults requires building a culture that challenges age-based assumptions about insulation from sexual risk and expands STI prevention efforts to more meaningfully include *anyone* experiencing sex with a new partner, regardless of their age.

**Key messages**

- Midlife men and women with new sexual partners experience multiple constraints on their navigation of STI risk and prevention.
- STI prevention services and campaigns geared towards younger people may not adequately address these age-specific factors.
- Efforts to reduce and prevent STI transmission in midlife should extend beyond increasing individual knowledge and address sexual risk and vulnerability among midlife men and women in the broader context of their lives and social networks.
