# Supplementary material for: Navigating new sexual partnerships in midlife: a socioecological perspective on factors shaping STI risk perceptions and practices
Source: Sex Transm Infect. 2020 Feb 10;96(4):238–45. doi: 10.1136/sextrans-2019-054205 (PMC7279207; doi:10.1136/sextrans-2019-054205)
Supplement: Supplementary data [file sextrans-2019-054205supp001.pdf]

Web Appendix Table 1: Sexual risk perceptions, partnerships and practices among midlife men and women reporting new opposite-sex partners in the past year (Natsal-3)

**Denominator: Men & women aged 40-59yrs who reported 1+ new opposite sex partner(s), past year**	**Men**	**Women**
Unweighted N	*241*	*242*
Weighted N	*320*	*241*
	% [95% CI]	% [95% CI]
Considers self to be at 'not at all at risk' of STI	50.3% [43.6, 51.0]	60.2% [53.1, 66.9]
Considers self to be at 'not at all at risk' of HIV	51.9% [44.7, 59.1]	61.9% [54.8, 68.5]
Total number of opposite-sex partners, past year		
1	41.0% [34.0, 48.4]	57.4% [50.2, 64.4]
2	24.4% [19.0, 30.7]	25.5% [19.9, 32.2]
3-4	23.1% [17.5, 29.7]	11.7% [8.0, 16.8]
5+	11.5% [7.5, 17.3]	5.3% [2.9, 9.6]
Had condomless sex with 1+ new partner*, past year	71.0% [64.2, 77.0]	72.1% [65.5, 77.9]
Concurrent (overlapping) sexual partners, past *five* years	38.5% [31.7, 45.8]	21.9% [16.6, 28.3]
Attended a sexual health clinic, past year	5.5% [2.9, 10.2]	4.8% [2.6, 8.7]

* Defined as reporting condomless sex at first sex with any of the (max.) 3 most recent partners (if in the past year) and/or reported 1+ new partner(s) and no condom use in the past year
